# *Cassia tora* L. (Jue-ming-zi) has anticancer activity in TCA8113 cells *in vitro* and exerts anti-metastatic effects *in vivo*

**DOI:** 10.3892/ol.2012.1097

**Published:** 2012-12-28

**Authors:** XIN ZHAO, QIANG WANG, YU QIAN, LIANG PANG

**Affiliations:** 1 Department of Biological and Chemical Engineering, Chongqing University of Education, Chongqing 400067;; 2 Department of Oral and Maxillofacial Surgery, the Affiliated Hospital of Stomatology, Chongqing Medical University, Chongqing 401147, P.R. China

**Keywords:** *Cassia tora* L., anticancer, apoptosis, anti-metastasis

## Abstract

*Cassia tora* L. (Jue-ming-zi) is a traditional Chinese medicine widely used in East Asia. The *in vitro* anticancer effects of Jue-ming-zi were evaluated in TCA8113 human tongue carcinoma cells using a 3-(4,5-dimethyl-2-thiazolyl)-2,5-diphenyltetrazolium bromide (MTT) assay. At a concentration of 1.0 mg/ml, *Cassia tora* L. inhibited the growth of TCA8113 cells by 72%; this inhibiton was greater than that by 0.5 and 0.25 mg/ml *Cassia tora* L. (43 and 16%, respectively). To elucidate the inhibitory mechanisms underlying the anticancer effect of *Cassia tora* L. in cancer cells, the expression of genes associated with apoptosis, inflammation and metastasis were measured using RT-PCR and western blot analysis. *Cassia tora* L. significantly induced apoptosis in cancer cells (P<0.05) by upregulating Bax, caspase-3 and caspase-9, and by downregulating Bcl-2. The expression of genes associated with inflammation, including NF-κB, iNOS and COX-2, was significantly downregulated (P<0.05) by *Cassia tora* L., demonstrating its anti-inflammatory properties. *Cassia tora* L. also exerted a significant anti-metastatic effect on cancer cells as demonstrated by decreased mRNA expression of matrix metalloprotease (MMP) genes and increased expression of tissue inhibitors of metalloproteinases (TIMPs), and as confirmed by the inhibition of induced tumor metastasis induced in 26-M3.1 colon cells in BALB/c mice. Our results demonstrated that *Cassia tora* L. exhibited the most potent *in vitro* anticancer effects, induced apoptosis, had anti-inflammatory activities and exerted *in vivo* anti-metastatic effects. Additionally, the anticancer, anti-inflammatory and anti-metastatic effects of the higher *Cassia tora* L. concentrations were stronger compared with those of the lower *Cassia tora* L. concentrations tested.

## Introduction

The Chinese herb, Jue-ming-zi, is the seed of the plant *Cassia tora* L. (Leguminosae), and has been used as a laxative and a tonic, as well as being a popular health tea drink. The commercial products of *Cassia tora* L. include both unroasted and roasted samples, and the laxative effect was found to be higher in unroasted compared with roasted *Cassia tora* L samples (1).

Pharmaceutical research has concentrated on the beneficial activities of *Cassia tora* L. such as its liver-protection, anti-aging, anticancer and antioxidant effects (2–5). *Cassia tora* L. contains anthraquinones, naphtho-pyrones, fatty acids, amino acids and inorganic elements (6). Types of *Cassia tora* L. with a high anthraquinone content, such as chrysophanol, physcion and obtusin, may help to decrease blood lipid levels (7).

The induction of apoptosis in cancer cells is initially identified by morphological changes including cell shrinkage, membrane blebbing, chromatin condensation and nuclear fragmentation (8). Apoptosis is an important defense against cancer. The process involves the elimination of potentially harmful cells. Many diseases have been associated with dysregulated apoptotic processes, ultimately leading to the inhibition of cell death and the propagation of diseases such as cancer (9).

Caspases are central components of the apoptotic response. Caspase-9 is an apical isoform involved in mitochondria-dependent apoptosis. This factor primarily activates caspase-3, which then serves as a gateway for the activation of downstream caspases (10). Nuclear factor-κB (NF-κB) is involved in the inhibition of apoptosis, stimulation of cell proliferation, inflammation, immune response and tumorigenesis. Activation of NF-κB generally prevents apoptosis. Expression of inducible nitric oxide synthase (iNOS) and cyclooxygenase (COX)-2, two genes regulated by NF-κB, are induced by inflammation and are frequently overexpressed in cancer cells. Increased NF-κB activity that is localized in the nucleus is particularly found in cells where there is abundant expression of iNOS and COX-2 (11).

A previous epidemiological study demonstrated that chronic inflammation predisposes individuals to various types of cancer (12). Hallmarks of inflammation-related cancers include the presence of inflammatory cells and mediators in tumor tissues, tissue remodeling and angiogenesis similar to that seen during chronic inflammatory responses, and tissue repair. The study of mechanisms underlying inflammation-related cancer has focused on the early stages of cancer; however, inflammatory mediators and cells are also involved in the migration, invasion and metastasis of malignant cells (13). Metastasis is the leading cause of mortality among cancer patients, and involves the spread of cancer from a primary site and the formation of new tumors in distant organs. Matrix metalloproteases (MMPs) are important in numerous physiological and pathological processes including embryonic development, morphogenesis, reproduction, tissue remodeling, arthritis, cardiovascular disease and metastasis (14). MMP activity is inhibited by specific endogenous tissue inhibitors of metalloproteinases (TIMPs) (15). To prevent the majority of cancer types, improved treatments for metastasis are required (16,17).

Previously, *Cassia tora* L. demonstrated strong *in vitro* anticancer effects in JTC-26 human cervical cancer cells (6). In the present study, we further examined the anticancer and anti-metastatic effects of *Cassia tora* L.; *Cassia tora* L. was administered to human tongue carcinoma TCA8113 cells and the molecular mechanisms underlying the anticancer effects of the *Cassia tora* L. were studied. Changes in activities of *Cassia tora* L. at different concentrations were evaluated and their anti-metastatic effects were assessed in mice with tumors propagated by 26-M3.1 colon carcinoma cells.

## Materials and methods

### Preparations of *Cassia tora* L

*Cassia tora* L. (Jue-ming-zi) was purchased from Yunnan Baiyao Group Co. Ltd. (Kunming, China) and stored at −80°C and freeze-dried to produce a powder. A 20-fold volume of methanol was added to the powdered sample and extracted twice by stirring overnight. The methanol extract was evaporated using a rotary evaporator (N-1100; Eywla; Tokyo, Japan), concentrated and then dissolved in dimethylsulfoxide (DMSO; Amresco, Solon, OH, USA) to adjust to the stock concentration (20%, w/v).

### Cancer cell preparation

Human tongue carcinoma TCA8113 cells obtained from the Shanghai Institute of Biochemistry and Cell Biology (SIBCB; Shanghai, China) were used in the experiments. The cells were cultured in Roswell Park Memorial Institute (RPMI)-1640 medium (Gibco Co., Birmingham, MI, USA) supplemented with 10% fetal bovine serum (FBS; Gibco Co.) and 1% penicillin-streptomycin (Gibco Co.), at 37°C in a humidified atmosphere containing 5% CO_2_ (model 311 S/N29035; Forma; Waltham, MA, USA). The medium was replaced two or three times each week.

### 3-(4,5-dimethyl-2-thiazolyl)-2,5-diphenyltetrazolium bromide (MTT) assay

The anticancer effects of *Cassia tora* L. were assessed by an MTT assay. Human tongue carcinoma TCA8113 cells were seeded in a 96-well plate at a density of 2×10^4^ cells/ml and a volume of 180 *μ*l/well. *Cassia tora* L. solution (20 *μ*l) was added at concentrations of 0.25, 0.5 and 1.0 mg/ml, and then the cells were incubated at 37°C in 5% CO_2_ for 48 h. An MTT solution (200 *μ*l; 5 mg/ml; Amresco) was added and the cells were cultured for a further 4 h under the same conditions. Following removal of the supernatant, 150 *μ*l DMSO was added to each well and mixed for 30 min. Subsequently, the absorbance of each well was measured with an enzyme-linked immunosorbant assay (ELISA) reader (model 680; Bio-Rad; Hercules, CA, USA) at 540 nm (18).

### Measuring RNA expression using reverse transcription-polymerase chain reaction (RT-PCR)

Total RNA was isolated from human tongue carcinoma TCA8113 cells using TRIzol reagent (Invitrogen Life Technologies; Carlsbad, CA, USA) according to the manufacturer’s instructions. The RNA was digested with RNase-Free DNase (Roche; Basel, Switzerland) for 15 min at 37°C and purified using an RNeasy kit (Qiagen; Hilden, Germany) according to the manufacturer’s instructions. cDNA was synthesized from 2 *μ*g of the total RNA by incubation at 37°C for l h with AMV reverse transcriptase (GE Healthcare, Little Chalfont, UK) and random hexanucleotides ,according to the manufacturer’s instructions. The sequences of the primers used to specifically amplify the genes of interest are listed in Table I. Amplification was performed in a thermal cycler (Eppendorf; Hamburg, Germany). The PCR products were separated in 1.0% agarose gels and visualized by ethidium bromide (EtBr) staining (19).

### Protein extraction and western blot analysis

Total cell lysates were obtained with an extraction buffer as previously described by Choi *et al*(20). Protein concentrations were determined using a protein assay kit (Bio-Rad). For western blot analysis, aliquots of the lysate containing 30–50 *μ*g of protein were separated by sodium dodecyl sulfate-polyacrylamide gel electrophoresis (SDS-PAGE) and then electrotransferred onto a nitrocellulose membrane (Schleicher and Schuell; Keene, NH, USA). The membranes were subjected to immunoblot analysis and the proteins were visualized by an enhanced chemiluminescence (ECL) method (GE Healthcare). The cell lysates were separated by 12% SDS-PAGE, transferred onto a polyvinylidene fluoride membrane (GE Healthcare), blocked with 5% skimmed milk and incubated with the primary antibodies (dilution, 1:1,000). Antibodies against Bax, Bcl-2, iNOS and COX-2 were obtained from Santa Cruz Biotechnology, Inc. (Santa Cruz, CA, USA). Following incubation with horse-radish peroxidase-conjugated secondary antibody at room temperature, immunoreactive proteins were detected using an ECL assay kit (GE Healthcare) according to the manufacturer’s instructions. Bands in the blot were visualized using a LAS3000 luminescent image analyzer (Fujifilm Life Science; Tokyo, Japan).

### Measuring lung metastasis following Cassia tora L. treatment in BALB/c mice bearing 26-M3.1 colon carcinoma cell tumors

26-M3.1 colon carcinoma cells were obtained from Professor Yoon, Department of Food and Nutrition, Yuhan University (Bucheon, South Korea). These highly metastatic cells were maintained as monolayers in EMEM (Gibco Co.) supplemented with 7.5% FBS, a vitamin solution, sodium pyruvate, non-essential amino acids and L-glutamine (Gibco Co.). The cultures were maintained in a humidified atmosphere of 5% CO_2_ at 37°C. Experimental lung metastasis was induced by injecting colon 26-M3.1 cells into the lateral tail vein of 6-week-old female Balb/c mice (Experimental Animal Center of Chongqing Medical University; Chongqing, China) (21). *Cassia tora* L. solution (50, 100 or 200 mg/kg) was subcutaneously injected into the mice, which were then intravenously inoculated with 26 M-3.1 cells (2.5×10^4^/mouse) after 2 days. The mice were sacrificed after 2 weeks and the lungs were fixed in Bouin’s solution (saturated picric acid: formalin: acetic acid; 15:5:1; v/v/v). The rate of metastasis was assessed by counting the number of lung tumor colonies using a digital camera (Canon D550; Canon, Inc.; Tokyo, Japan). protocol for the animal experiments was approved by the Animal Ethics Committee of Chongqing Medical University.

### Statistical analysis

Data are presented as the mean ± standard deviation. Differences in the mean values of individual groups were assessed with a one-way analysis of variance (ANOVA) with a Duncan’s multiple range test. P<0.05 was considered to indicate a statistically significant difference. SAS software, version 9.1 (SAS Institute Inc.; Cary, NC, USA) was used for statistical analyses.

## Results

### In vitro anticancer effect of *Cassia tora* L. on TCA8113 cells

The anticancer effect of *Cassia tora* L. on TCA8113 cells was evaluated using an MTT assay. The growth inhibitory rates of human tongue carcinoma TCA8113 cells treated with the different concentrations of *Cassia tora* L. are demonstrated in Table II. When *Cassia tora* L. solution was administered to TCA8113 cells, the growth inhibitory rates observed with concentrations of 0.25, 0.5 and 1.0 mg/ml were 16, 43 and 72%, respectively (P<0.05). These results demonstrated that *Cassia tora* L. has a significant anti-proliferative effect on TCA8113 cells. In addition, the higher the concentration of *Cassia tora* L., the stronger the anticancer effect.

### Apoptosis-related gene expression of Bax, Bcl-2 and caspases

**Figure 1:**
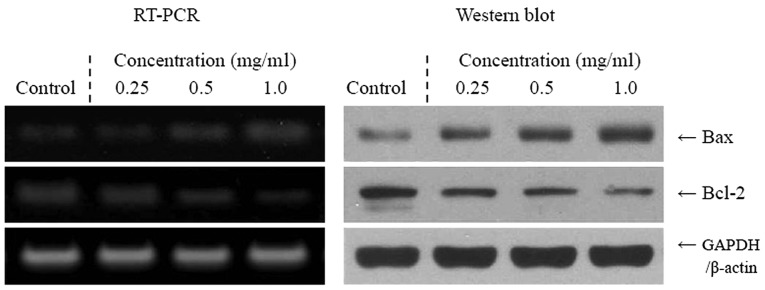
Effects of *Cassia tora* L. on the mRNA and protein expression of Bax and Bcl-2 in human tongue carcinoma TCA8113 cells.

**Figure 2:**
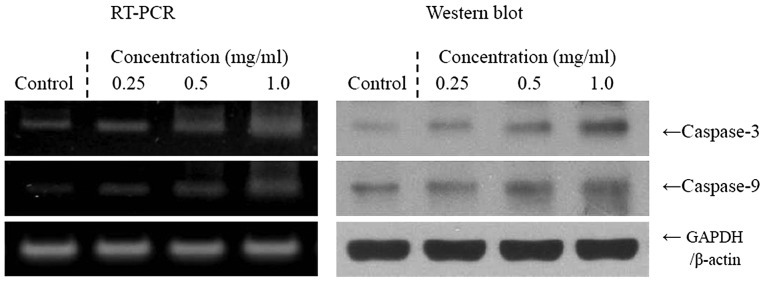
Effects of *Cassia tora* L. on the mRNA and protein expression of caspase-3 and -9 in human tongue carcinoma TCA8113 cells.

To elucidate the mechanisms underlying the inhibition of cancer cell growth by the *Cassia tora* L., the expression of Bax, Bcl-2, and caspase-3 and -9 was measured in human tongue carcinoma TCA8113 cells by RT-PCR and western blot analyses after a 48-h incubation with different concentrations of *Cassia tora* L. solution. As demonstrated in Fig. 1, the expression of pro-apoptotic Bax and anti-apoptotic Bcl-2 demonstrated significant changes in the presence of 1.0 mg/ml *Cassia tora* L. These results suggest that *Cassia tora* L. induced apoptosis in the TCA8113 cells via a Bax- and Bcl-2-dependent pathway. The mRNA and protein expression levels of caspase-3 and -9 were very low in untreated control TCA8113 cells, but significantly increased following treatment of the cells with 1.0 mg/ml *Cassia tora* L. Caspase-3 and -9 mRNA and protein expression was gradually elevated by treatment with increased *Cassia tora* L. concentrations (Fig. 2). More specifically, the induction of apoptosis by *Cassia tora* L. was correlated with the upregulation of Bax, caspase-3 and -9, and the downregulation of Bcl-2, in terms of mRNA and protein expression. The effects of 1.0 mg/ml *Cassia tora* L. were greater compared with those of 0.25 and 0.5 mg/ml *Cassia tora* L.

### Inflammation-related gene expression of NF-κB, IκB-α, iNOS and COX-2

**Figure 3:**
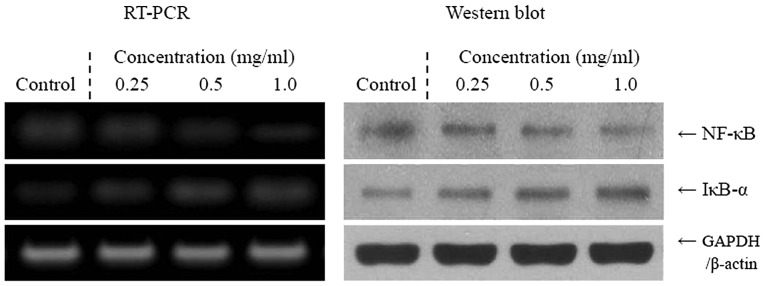
Effects of *Cassia tora* L. on the mRNA and protein expression of NF-κB and IκB-α in human tongue carcinoma TCA8113 cells.

**Figure 4:**
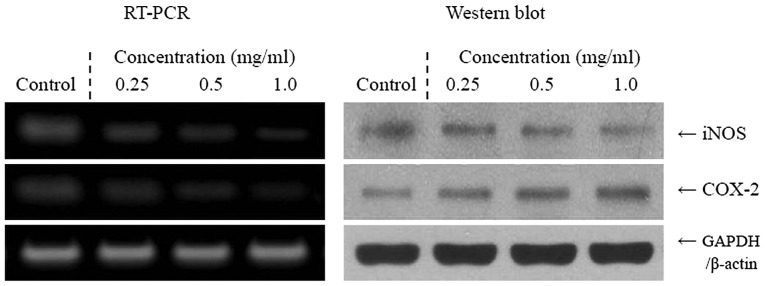
Effects of *Cassia tora* L. on the mRNA and protein expression of iNOS and COX-2 in human tongue carcinoma TCA8113 cells.

We determined whether the anticancer effects of *Cassia tora* L. were correlated with the inhibition of NF-κB, IκB-α, iNOS and COX-2 gene expression. As demonstrated in Fig. 3, mRNA and protein expression of NF-κB and IκB-α were reduced in TCA8113 cells treated with 1.0 mg/ml *Cassia tora* L. solution. *Cassia tora* L. significantly modulated the expression of genes associated with inflammation. The mRNA and protein expression of NF-κB decreased while IκB-α mRNA levels increased. Additionally, the mRNA and protein expression of COX-2 and iNOS gradually decreased in the presence of the *Cassia tora* L., depending on the concentration (Fig. 4). Our findings indicate that *Cassia tora* L. may help to prevent cancer in the early stages by increasing anti-inflammatory activities. Overall, the results of this experiment demonstrate that the higher concentration of *Cassia tora* L. had a stronger anti-inflammatory effect on the tongue carcinoma cells than the lower concentrations tested.

### Metastasis-related MMP and TIMP gene expression

**Figure 5:**
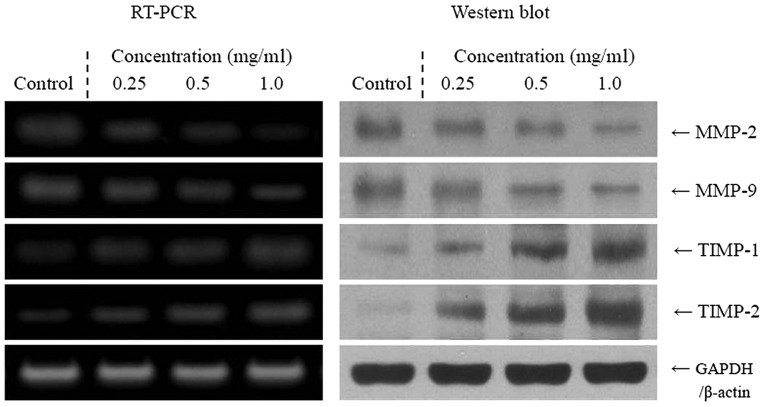
Effects of *Cassia tora* L. on the mRNA and protein expression of matrix metalloproteases (MMPs) and tissue inhibitors of metalloproteinases (TIMPs) in human tongue carcinoma TCA8113 cells.

RT-PCR and western blot analyses were conducted to determine whether the anti-metastatic effect of *Cassia tora* L. was due to gene regulation of metastatic mediators, specifically MMPs (MMP-2 and -9) and TIMPs (TIMP-1 and -2), in TCA8113 cells. As demonstrated in Fig. 5, 1.0 mg/ml *Cassia tora* L. significantly decreased the mRNA and protein expressions of MMP-2 and -9, while it increased the expression of TIMP-1 and -2. These changes in TIMP and MMP expression resulting from *Cassia tora* L. treatment effectively led to metastatic inhibition *in vitro*. Our results also demonstrated that the higher concentration of *Cassia tora* L. had a stronger anti-metastatic activity than the lower concentrations of *Cassia tora* L. tested.

### In vivo anti-metastatic effect of *Cassia tora* L

**Figure 6:**
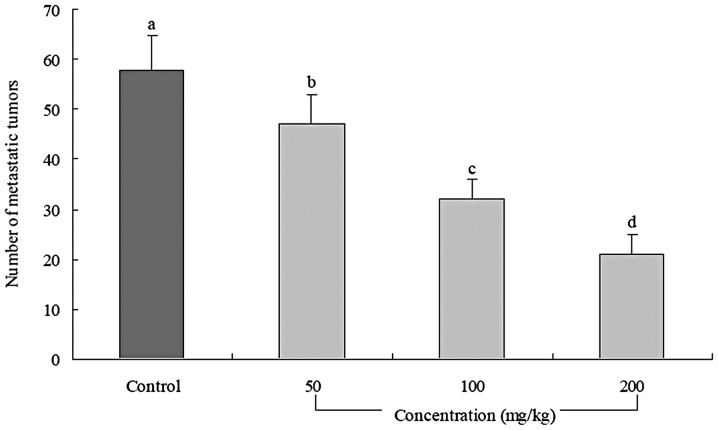
Inhibitory effect of *Cassia tora* L. on the metastasis of tumors produced by colon 26-M3.1 cells in Balb/c mice. The mice were administered the indicated dose of *Cassia tora* L. by subcutaneous injection and then inoculated intravenously with 26 M-3.1 cells (2.5×10^4^ cells/mouse) after 2 days. Eight mice were assigned to each group 14 days after intravenous injection of the cancer cells. Lungs were removed and the number of lung tumor colonies were counted. ^a–d^Mean values with different letters over the bars are significantly different (P<0.05) according to Duncan’s multiple range test.

Prophylactic inhibition of tumor metastasis by *Cassia tora* L. was evaluated using an experimental mouse metastasis model (Fig. 6). All mice treated with *Cassia tora* L. had significantly fewer lung metastatic colonies compared with the control mice (number of metastatic tumors, 58±7; number in each group=10; P<0.05). *Cassia tora* L. was most effective at inhibiting lung metastasis at a concentration of 200 mg/kg. This concentration (inhibitory rate, 64%; number of metastatic tumors, 21±4) inhibited tumor formation and lung metastasis to a greater degree than the 100 mg/kg solution (inhibitory rate, 45%; number of meta-static tumors, 32±4) or the 50 mg/kg solution (inhibitory rate, 19%; number of metastatic tumors, 47±6) of *Cassia tora* L.

## Discussion

Although *Cassia tora* L. has been used as a medicine, scientific data concerning its effects is limited. *Cassia tora* L. has previously been demonstrated to have various therapeutic effects on numerous pathological conditions such as inflammation, aging and cancer (3,4,6).

Apoptosis is a fundamental cellular event, and understanding its mechanisms of action will aid in the exploitation of this process in tumor diagnosis and therapy (22). In a healthy cell, the anti-apoptotic protein Bcl-2 is expressed on the outer mitochondrial membrane surface (23). As Bax and Bcl-2 genes are mainly expressed during apoptosis, we determined that these genes regulate apoptotic activity. Apoptosis results from the activation of caspase family members that act as aspartate-specific proteases (24). Caspases form a proteolytic network within the cell whereby upstream initiator caspases (such as caspase-9) are activated early on in the apoptotic process and in turn activate other downstream caspases (such as caspase-3). Cytochrome *c* and procaspase-9 processing are highly dependent on caspase-3, placing this caspase in a central position as a regulator of essential apoptotic pathways in cancer cells (25). Caspase-3 has also been demonstrated to be involved in the amplification of apoptotic signals by cleaving Bcl-2 (26).

Additionally, anticancer mechanisms underlying the effect of *Cassia tora* L. on human cancer cells involve the induction of apoptosis by increasing the number of apoptotic bodies, regulating the mRNA and protein expression of Bax and Bcl-2, and promoting anti-inflammatory effects by down-regulating iNOS and COX-2 gene expression. COX-2 has been suggested to be important in colon carcinogenesis, while NOS, along with iNOS, may be a good target for the chemoprevention of colon cancer (27). NF-κB is one of the most ubiquitous transcription factors, and it regulates the expression of genes required for cellular proliferation, inflammatory responses and cell adhesion (28). NF-κB is present in the cytosol where it is bound to the inhibitory protein, IκB. Following its induction by a variety of agents, NF-κB is released from IκB and trans-locates to the nucleus where it binds to the κB binding sites in the promoter regions of target genes (29). These mechanisms may be involved in the anticancer effects of *Cassia tora* L. in tongue carcinoma cells. Based on the results of the MTT assay and the expression patterns of pro-apoptotic genes observed in the present study, we conclude that cancer cells treated with *Cassia tora* L. underwent apoptosis. The anticancer effects of *Cassia tora* L. in JTC-26 human cervical cancer cells were evaluated in a previous study by an MTT assay and RT-PCR or western blot analysis, and were similar to our findings (6).

Metastasis is defined as the spread of cancer cells from one organ or area to an adjacent organ or another location (30,31). Malignant tumor cells are considered to have the capacity to metastasize. Cancer occurs when the cells in a tissue have been genetically damaged in a progressive manner, resulting in cancer stem cells that possess a malignant phenotype. When the tumor cells have come to rest in another site, they penetrate the vessel walls, continue to multiply and eventually form another tumor.

MMPs, a family of zinc-dependent endopeptidases, are important in tumorigenesis and metastasis. MMPs are able to cleave almost all extracellular matrix (ECM) substrates. Degradation of the ECM is a key event in tumor progression, invasion and metastasis (32). Among the MMP family members, MMP-2 and -9 are important molecules for cancer invasion (33,34), and are highly expressed in breast and colon cancer cells (35–37). Inhibition of MMP activity is useful for controlling tumorigenesis and metastasis (38). TIMPs are naturally occurring inhibitors of MMPs which prevent catalytic activity by binding to activated MMPs, thereby blocking breakdown of the ECM (39). Disturbances in the ratio between MMPs and TIMPs have been observed during tumorigenesis (40). Maintaining the balance between MMPs and TIMPs, or increasing TIMP activity, are useful ways to control tumor metastasis (41). Experimental evidence demonstrating the role of MMPs in metastasis has been obtained by *in vitro* invasion assays and *in vivo* xenograft metastasis experiments.

MMP-2 and -9 are key factors in cancer cell invasion and metastasis both *in vivo* and *in vitro*(42). Spontaneous and experimental metastasis to the liver is decreased in mice over-expressing TIMP1, and increased in mice expressing antisense TIMP-1 mRNA (43). Ectopic overexpression of TIMP-1 in the brain of transgenic mice also reduces experimental metastasis to the brain (44). In particular, MMP-2 and -9 are important for tumor invasion and angiogenesis. Thus, tumor metastasis may be inhibited by blocking MMP synthesis and activity (45). Colon 26-M3.1 carcinoma cells have been used to evaluate anti-metastasis effects *in vivo*(46).

In the current study, different concentrations of *Cassia tora* L. were employed in our experiments. *Cassia tora* L. exerted anticancer and anti-metastatic effects on TCA8113 cells. All concentrations of *Cassia tora* L. were found to have *in vitro* anti-metastasis effects based on the RT-PCR and protein analysis of MMP and TIMP gene expression, and also showed anti-metastasis effects *in vivo*. Further research is required to explain the mechanisms associated with these effects.

In summary, various *in vitro* experimental methods, including MTT, RT-PCR and western blot analysis, were employed to evaluate the anticancer effects of *Cassia tora* L. A mouse model bearing tumors produced by 26-M3.1 colon carcinoma cells was used to study the *in vivo* effects of *Cassia tora* L. Overall, *Cassia tora* L. demonstrated potent *in vitro* and *in vivo* anticancer activities, particularly in combating *in vivo* tumor metastasis. The functional contents of *Cassia tora* L. are important for augmenting these anti-cancer effects. A high concentration of *Cassia tora* L. solution increased the anticancer properties in the present study. The active compounds resulting from *Cassia tora* L. require identification and evaluation in future studies.

## Figures and Tables

**Table I:** Sequences of reverse transcription-polymerase chain reaction (RT-PCR) primers used in this study.

Gene name	Sequence
Bax	Forward: 5′-AAG CTG AGC GAG TGT CTC CGG CG-3′ Reverse: 5′-CAG ATG CCG GTT CAG GTA CTC AGT C-3′
Bcl-2	Forward: 5′-CTC GTC GCT ACC GTC GTG ACT TGG-3′ Reverse: 5′-CAG ATG CCG GTT CAG GTA CTC AGT C-3′
Caspase-3	Forward: 5′-CAA ACT TTT TCA GAG GGG ATC G-3′ Reverse: 5′-GCA TAC TGT TTC AGC ATG GCA-3′
Caspase-9	Forward: 5′-GGC CCT TCC TCG CTT CAT CTC-3′ Reverse: 5′-GGT CCT TGG GCC TTC CTG GTA T-3′
NF-κB	Forward: 5′-CAC TTA TGG ACA ACT ATG AGG TCT CTG G-3′ Reverse: 5′-CTG TCT TGT GGA CAA CGC AGT GGA ATT TTA GG-3′
IκB-α	Forward: 5′-GCT GAA GAA GGA GCG GCT ACT-3′ Reverse: 5′-TCG TAC TCC TCG TCT TTC ATG GA-3′
iNOS	Forward: 5′-AGA GAG ATC GGG TTC ACA-3′ Reverse: 5′-CAC AGA ACT GAG GGT ACA-3′
COX-2	Forward: 5′-TTA AAA TGA GAT TGT CCG AA-3′ Reverse: 5′-AGA TCA CCT CTG CCT GAG TA-3′
MMP-2	Forward: 5′-CTT CTT CAA GGA CCG GTT CA-3′ Reverse: 5′-GCT GGC TGA GTA CCA GTA-3′
MMP-9	Forward: 5′-TGG GCT ACG TGA CCT ATG AC-3′ Reverse: 5′-GCC CAG CCC ACC TCC ACT CC-3′
TIMP-1	Forward: 5′-GTC AGT GAG AAG CAA GTC GA-3′ Reverse: 5′-ATG TTC TTC TCT GTG ACC CA-3′
TIMP-2	Forward: 5′-TGG GGA CAC CAG AAG TCA AC-3′ Reverse: 5′-TTT TCA GAG CCT TGG AGG AG-3′
GAPDH	Forward: 5′-CGG AGT CAA CGG ATT TGG TC-3′ Reverse: 5′-AGC CTT CTC CAT GGT CGT GA-3′

**Table II:** Growth inhibition of human tongue carcinoma TCA8113 cells by different concentrations of *Cassia tora* L., as evaluated by a 3-(4,5-dimethylthiazol-2-yl)-2,5-diphenyltetrazolium bromide (MTT) assay.

	OD540 (concentration of sample, mg/ml)		
Treatment	0.25	0.5	1.0
Control (untreated)		0.497±0.005^a^	
*Cassia tora* L.	0.417±0.008^b^(16)	0.283±0.010^c^(43)	0.139±0.007^d^(72)

The values in parentheses are the inhibition rates (%).

a–d Mean values with different letters in the same column are significantly different (P<0.05) according to the Duncan’s multiple range test.
